# High-throughput screening of the static friction and ideal cleavage strength of solid interfaces

**DOI:** 10.1038/s41598-019-49907-2

**Published:** 2019-11-19

**Authors:** Michael Wolloch, Gabriele Losi, Mauro Ferrario, M. Clelia Righi

**Affiliations:** 1 Dipartimento di Scienze Fisiche, Informatiche e Matematiche, Università di Modena e Reggio Emilia, Via Campi 213/A, 41125 Modena, Italy; 2 Faculty of Physics, University of Vienna, Währinger Strasse 17, 1090 Vienna, Austria; 3 CNR-Institute of Nanoscience, S3 Center, Via Campi 213/A, 41125 Modena, Italy

**Keywords:** Theory and computation, Mechanical properties, Surfaces, interfaces and thin films, Mechanical properties

## Abstract

We present a comprehensive *ab initio*, high-throughput study of the frictional and cleavage strengths of interfaces of elemental crystals with different orientations. It is based on the detailed analysis of the adhesion energy as a function of lateral, *γ*(*x*, *y*), and perpendicular displacements, *γ*(*z*), with respect to the considered interface plane. We use the large amount of computed data to derive fundamental insight into the relation of the ideal strength of an interface plane with its adhesion. Moreover, the ratio between the frictional and cleavage strengths is provided as good indicator for the material failure mode – dislocation propagation versus crack nucleation. All raw and curated data are made available to be used as input parameters for continuum mechanic models, benchmarks, or further analysis.

## Introduction

High-throughput studies have recently become a valuable tool for material advancements, which are of tremendous importance for many industrial applications and closely tied to many societal challenges like clean energy production. The cost and trouble of experimentally testing the vast number of possible compounds and alloys which could potentially have optimal properties for selected applications is often prohibitive. However, the exponential growth of computer power and the development of efficient data mining and curating techniques using machine learning approaches have opened a new avenue for large scale computational materials research through high-throughput screening^1–4^. We aim to use this relatively new approach to investigate solid interfaces, and screen figures of merit important for tribological applications.

Tribological problems become relevant whenever materials come into contact while moving laterally to each other. Tribology is the science of adhesion, friction, wear, and lubrication, to date largely dominated by the experimental community. In the last decades, however, simulations of tribological systems have become more and more common^5^. Due to surface roughness tribological contacts are typically nanoscopic in size^6,7^, even for macroscopic bodies, so it is meaningful to use atomistic models^8–13^, and even employ accurate quantum methods to describe the interatomic interactions^14–20^. If quantum methods like Density Functional Theory (DFT) are used, it is possible to extract fundamental properties of the investigated materials without any prior knowledge, which in turn dictate the adhesive properties, the frictional response, the ductility (which ultimately governs the mode of failure of the material), and more.

We have recently introduced high throughput computations in tribology^21^, which addresses the problem of choosing the right materials, coatings, and lubricants for a specific task from a new angle: simulate a large number of different interfaces in a high-throughput manner and choose the most promising to be tested in the laboratory. In the present paper we will apply an improved and extended version of our previously described workflow^21^, which is designed to construct and screen solid-solid interfaces. Our computational setup is based on the protocol described in ref.^16^ for the calculation of the intrinsic resistance to sliding of a solid interface from first principles. The protocol is based on the calculation of the *γ*-surface, also called generalized stacking fault energy or potential energy surface (PES), which describes the variation of the adhesion energy between two surfaces (modelled by two mating slabs within the same supercell) as a function of their relative lateral position. This energy variation is the origin of frictional forces. We obtain the ideal or theoretical shear strength (which we abbreviate also as *τ* in figures and tables), as the static friction force per unit area of the interface, i.e., its resistance to a shear load, as the maximum restoring force calculated by the PES derivative along the shear direction. In the same way we obtain the cleavage strength *σ*_*C*_ of the interface, i. e., its resistance to brittle fracture under tensile strain, from the derivative of the adhesion energy as a function of the surface separation.

While this protocol can be applied to obtain the ideal shear and cleavage strengths of generic interfaces, e.g., interfaces obtained by mating surfaces of different materials, or partially covered by adsorbates, here we focus on homogeneous interfaces formed by two equivalent surfaces. Thus we are effectively discussing the plastic deformation in bulk crystals known as “slip”. It should be noted that *τ* of homogeneous interfaces here calculated is related, but can differ, from the shear strength calculated by continuously straining the bulk unit cell and monitoring the resulting stresses. It has been shown that the generalized stacking fault energy differs by the affine strain energy in materials where the sliding of a layer is strongly coupled with the sliding of the adjacent layers, e.g., in materials with directional bonds^14^.

The ratio of the ideal shear strength and the cleavage strength *τ*/*σ*_*C*_ is also of interest to describe the failure characteristic of a material, since it is a quantitative indication of brittle versus ductile behaviour. It should be also noted that the response of materials and interfaces to external stresses is often strongly dependent on the creation and movement of dislocations^22^. Indeed the PES data we compute, is intimately related to the probability of dislocation nucleation, and can be used to connect results from dislocation mechanics with those from ab-initio calculations^23–26^.

In the following section we will present computations of the adhesion energy, (*γ*), the ideal shear strength, (*τ*), and the ideal cleavage strength under tensile load (*σ*_*C*_) for over 100 interfaces of 45 elemental crystals across the periodic table.

It has to be noted that the calculated material data are “ideal” as they are calculated for defect- and impurity-free, perfect crystals at zero temperature. Therefore the computed values have to be seen as upper bounds of those experimentally measured. Their knowledge is useful for defining *intrinsic* properties that can be used to readily make comparisons among different materials and fix their range of applicability. Moreover, measurements on near perfect mono-crystalline fibres (whiskers) are found to closely approach the ideal tensile strength values predicted by simulations^27^. In the same spirit, nanoindentation experiments report shear strengths close to the ideal values^28^. In these experiments the contact between the tip and the substrate is so small that only a single grain of material is probed and the chances are high that there are no preformed dislocations directly below the tip.

Another important application of ab-initio computed material properties is their use as input parameters for atomistically-informed continuum models. For example, the adhesion energy between two ideally flat planes (as calculated by our workflow) is a crucial parameter for the analysis of rough contacts on the nanoscale^29,30^ and can also be employed in complex models for the description of frictional hysteresis loops^31^. Similarly, the ideal cleavage strength is an important parameter for crack propagation models^32^, and the ideal shear strength is, for instance, needed for multiscale approaches to nanoindentation modelling^33^. The 0 K results presented in the following could also be used as the basis for further investigations at elevated temperatures. As a starting point the simple approximation of Kelly and Macmillan *τ*(*RT*) ∼ 0.4*τ*(0) could be used^34^.

## Results

We have used our workflow to calculate interfacial properties of 106 different interfaces of 44 elemental crystals. Mostly these are main group metals (and a few lanthanides), but we also include 9 different interfaces of two semiconductors, Si and Ge, crystallizing in the diamond structure, as well as three interfaces of *α*-tin, which shows the diamond structure as well. All raw data, including optimized lattice parameters, surface energies and *γ*-surface data will be accessible through provided supplementary data files (see Data availability section), while a table with the most important curated results (lattice parameter, *γ*, $\tau$, and *σ*_*C*_) is presented in the supplementary information as well.

### Potential energy surfaces, adhesion, and shear stress

**Figure 1:**
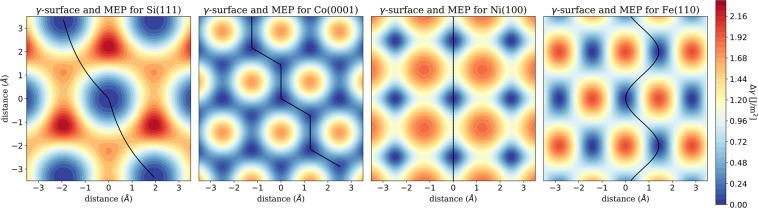
PES and MEP for four different lattice structures. All plots use the same scales and the minimum energy paths are shown in black.

First we look at the potential energy surface (PES or *γ*-surface), which allows to derive several important characteristics of an interface. The *γ*-surface represents the adhesive energy as a function of lateral displacement between the two surfaces. We make a total energy calculations at carefully selected points *S*(*x*_*i*_, *y*_*j*_) across the unit cell, making sure that all high symmetry position are included and the cell is sampled uniformly. During these calculations the atoms in the slabs are fixed in the coordinates parallel to the interface (*x*, *y*), but are free to move perpendicular to it (*z*). Radial basis functions are used for the interpolation between the computed data to obtain a smooth *γ*-surface, and the PES is shifted by subtracting the minimum energy (which corresponds to the adhesion energy *γ*), so that the maximal corrugation Δ*γ* is easily sighted as the highest peak of the *γ*-surface. In Fig. 1 four examples of different *γ*-surfaces are presented. The (111) interface of silicon in the cubic diamond structure, the (100) interface of fcc nickel, the (110) interface of bcc iron and the (0001) interface of hcp cobalt. They all show comparable corrugation Δ*γ* between 1.8 and 2.2 J/m^2^, with Co exhibiting the lowest and Si the highest Δ*γ*. Also shown in Fig. 1 are the associated minimum energy paths (MEPs), which connect the minima of the *γ*-surfaces by traversing the saddle points. They are computed automatically by application of the improved simplified string method from E, Ren and Vanden-Eijnden^21,35^. Once sliding is initiated, the MEP will be the path with the highest statistical weight. The maximal slope encountered by traversing the *γ*-surface along the MEP is assumed as the ideal shear strength of the interface, $\tau ={\tau }^{{\rm{M}}{\rm{E}}{\rm{P}}}$. The values of the maximal slopes along two perpendicular high symmetry directions, as well as their ratio are also reported as supplementary information to provide information on the friction anisotropy.

While Δ*γ* of the materials in Fig. 1 is comparable, the ideal shear strength along the MEPs differs by more than a factor two from $\tau ({\rm{C}}{\rm{o}})=6.95\,{\rm{G}}{\rm{P}}{\rm{a}}$ to $\tau ({\rm{N}}{\rm{i}})=14.57\,{\rm{G}}{\rm{P}}{\rm{a}}$. While the potential corrugation of the Ni(100) interface is the second smallest of the four interfaces depicted in Fig. 1 at 1.9 J/m^2^, its ideal shear strength is the largest. Obviously the barrier height along the MEP plays a more significant role than the total potential corrugation Δ*γ*, but while Si(111) has a 36% higher barrier than Ni(100), the ideal shear strength is nearly 9% lower. Of course this is routed in the different spacing of the minima and saddle points on the PESs. While the distance between the total minimum and the saddle point is ∼2 Å for Si(111), it is only ∼1.2 Å for Ni(100). This analysis shows the importance of a detailed investigation of the shape of the PES, and its derivative along selected paths.

**Figure 2:**
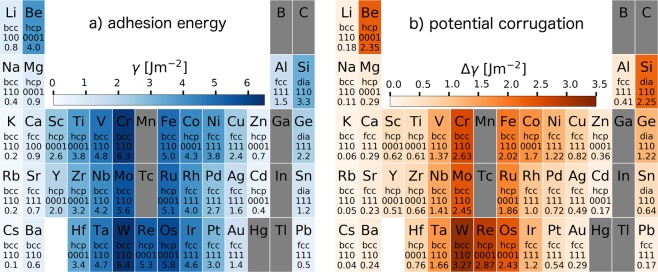
Values found for the adhesion *γ* (**a**), and the total corrugation of the *γ*-surface Δ*γ* (**b**), for the surface plane where they are minima, the values obtained for the other considered planes are reported as SI.

In Fig. 2 we plot two copies of the periodic table coloured with respect to the adhesion (note that we define *γ* in the intuitive way, so that a larger value corresponds to a stronger adhesion.) (a), and the corrugation of the *γ*-surface Δ*γ* (b). For all panels we show the lowest values we have found among the three lattice planes considered for each crystal (excluding hcp, where only the 0001 plane is considered), and indicate the corresponding plane as well as the crystal structure in the table.

**Figure 3:**
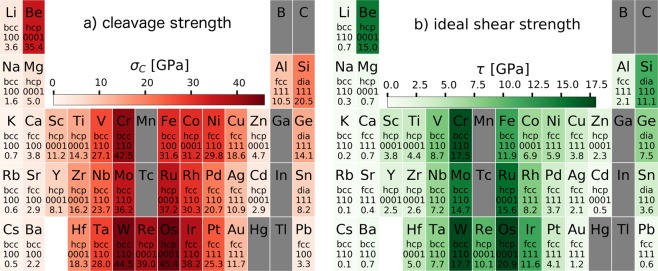
Values found for the cleavage strength *σ*_*C*_ (**a**) the shear strength along the MEP $\tau$ (**b**), for the slip plane where they are minima, the values obtained for the other considered planes are reported as SI.

The cleavage strength *σ*_*C*_ (a) and the ideal frictional strength along the MEP $\tau$ (b) are shown in Fig. 3. To the best of our knowledge, this is the first systematic analysis of ideal strength strength of elemental solids published so far. The procedure adopted to calculate *σ*_*C*_ will be explained in detailed in the following section.

**Figure 4:**
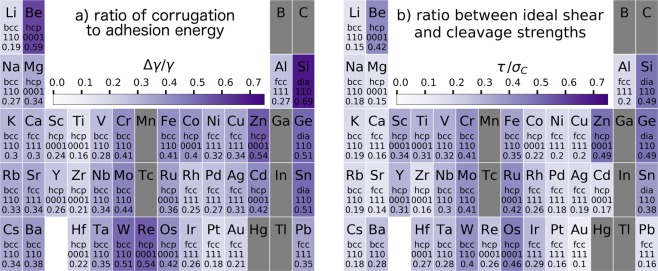
Ratios of the figures of merit presented in Figs 2(a) and 3(b), for the surface plane where they are minima. These ratios are measures of the ductility of the interface.

**Figure 5:**
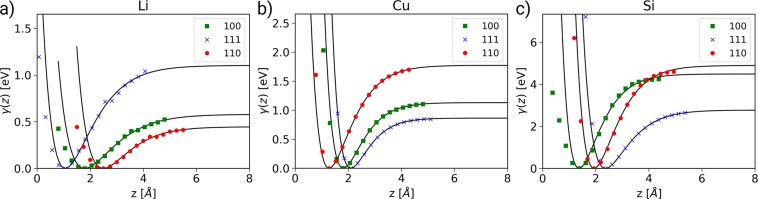
Examples for the fit quality of the UBER relations for bcc Li (**a**), fcc Cu (**b**), and Si (**c**) which crystallizes in the diamond structure.

The scales of Figs 2 and 3 are different, but the distribution of strong and light colours is very similar for all four subfigures. However, the interface planes where the minimal values for *γ*, $\tau$, and *σ*_*C*_ are found show some interesting patterns. In the case of the adhesion, bcc crystals show the lowest values for (110) surfaces, while crystals with fcc or diamond structures have the lowest values for the (111) surfaces. Notable exceptions are Li (bcc, 100, 9.8%), Ca (fcc, 100, 1.6%), and Si (diamond, 110, 2.1%), where the parentheses indicate lattice type, actual plane with minimal adhesion, and the percentage of adhesion increase with respect to the close packed plane. For Si, the difference in the adhesion is strongly dictated by the relaxation of the surfaces after separation, as also becomes apparent in Fig. 5(c), where they are not considered in the cleavage energy *E*_*C*_, and the small difference in *γ* between 111 and 110, becomes considerably larger.

For the ideal frictional strength, the data are even more homogeneous, with all bcc materials favouring the (110) interface and all fcc crystals the (111) plane. This is not a surprising result, since the bcc(110) and the fcc(111) are the closest packed interfaces of these crystal structures, where we expect them to show the least resistance to sliding. For all three materials in the diamond structure, the (110) plane shows the lowest and the (100) plane the largest ideal shear strength values. The trend of high frictional strength at the (100) interface continues for both fcc and bcc crystals but there are exceptions: Li, Rb, Ba, an Ta for bcc, where the (111) plane has the largest ideal shear strength, and Ir, Pt, Al, and Pb for fcc, where it is the (110) interface.

For the ideal cleavage strength *σ*_*C*_, which is discussed in more detail in the next section, there is a clear split for the bcc crystals. The group I and II elements exhibit the least resistance to cleavage normal to the (100) planes, whereas the bcc *d*-metals favour cleavage along the (110) plane, with Fe being the exception, also preferring the (100) plane. In the case of fcc, we observe three different groups: the *sp*-bonded simple metals Ca, Sr, and Pb cleave easiest at the (100) interface, while the noble metals, Pt, Cu, Ag, Au plus Al prefer the (111) direction. The remaining *d*-metals Rh, Ir, Ni, and Pd have the (110) interface as the preferred cleavage plane. The contrast to the behaviour of the cleavage strength with respect to the adhesion shows that the binding energy alone is not responsible for the cleavage strength, but the shape of the perpendicular potential profiles *γ*(*z*) is crucial (also see next section).

Since we have only calculated data of the basal (0001) plane for hcp systems to date, these systems have not been especially mentioned. However, we will include them in the discussion section when we analyze Figs 2 and 3 in more detail.

### Perpendicular potential profiles and cleavage strength

We have updated our workflow by fitting the perpendicular potential profiles *γ*(*z*), to the universal binding energy relation UBER (see e.g. ref.^36^),

1 $$\gamma (z)={E}_{C}[1-(1+\frac{z-{z}_{0}}{\ell })\exp (-\frac{z-{z}_{0}}{\ell })]\,,$$

where *E*_*C*_ is the cleavage energy in the minimum configuration, *z* is the distance between the slabs, *z*_0_ is their equilibrium distance (commonly set to 0), and $\ell$ is the critical length, at which *γ*(*z*) has an inflection point and the cleavage stress *σ*_*c*_ reaches a maximum. Due to the rigid separation model, the cleavage energy *E*_*C*_ is slightly larger than the adhesion *γ*, where relaxations of the separated slabs are considered.

Under tensile load (forces pulling at the two slabs normal to the interface, also called mode 1 loading) the maximal restoring force is realized at the inflection point and can be easily computed by taking the derivative of Eq. 1 with respect to *z*, and setting ${z}_{0}=0$ for simplicity and $z=\ell$ in the following,

2 $$\,\,\begin{array}{ccc}\frac{d\gamma }{dz}(z)=\frac{{E}_{C}}{{\ell }^{2}}z\,\exp (\frac{-z}{\ell }) & \to  & {\frac{d\gamma }{dz}|}_{z=\ell }=\frac{{E}_{C}}{\ell e}\,.\end{array}$$

In turn the ideal cleavage strength *σ*_*C*_ follows by dividing this force by the cell cross section *A*. Figure 2(b) shows the lowest calculated *σ*_*C*_ along with the corresponding interface plane for each considered element.

While separating the slabs perpendicular to the interface (starting in the global minimum of the *γ*-surface) we do not allow for any atomic relaxations, which is equivalent to the assumption that the interface fails in a perfectly brittle manner under tensile strain (mode 1 loading). This approach is commonly known as rigid body separation and more information about its application can be found in the Supplementary Information. It provides reliable upper limits for the ideal tensile strengths of materials^37^ and was shown to be well justified by comparison to a simulation of a crack tip under mode 1 loading^32^.

In Fig. 5 we plot three UBER fits alongside the computed DFT data to show the quality of the fits. Since we are primarily interested in the ideal strength of the material under tensile loading, the attractive regime, *z* > *z*_0_, is more important and we have used only those data points for fitting. This results in, sometimes significant, deviations of the fit to the data in the repulsive regime, as it can be seen for Li and Si in Fig. 5. However, the fits in the important attractive regime are excellent and the raw DFT data is available to the reader (see Data availability section) and can be fitted by cubic splines or other smooth functions to represent the whole interaction range^21,38^. Generally the quality of the fits are very high, with Li, Fig. 5(a), being one of the worst. In Fig. 3(b) we see the data for the lowest cleavage strength *σ*_*C*_ of each element, depending on the interface plane. We note that there are no long range dispersion interactions considered for this data, so the cleavage energy *E*_*C*_ and in turn the cleavage strength *σ*_*C*_ might be underestimated somewhat.

The corrugation Δ*γ*, and especially the ratio of the Δ*γ* to the surface energy *γ*/2 is an important measure for the ductility of a material, since it it is related to the relative probabilities of dislocation emission and crack propagation^39^. A periodic table for this ratio is given in Fig. 4(a), and it shows that indeed brittle materials (Si, Be, W, …) have a significantly higher Δ*γ/γ* ratio than ductile materials (Au, Pt, Ti, …). A similar result can be found if the ratio of the ideal shear to the cleavage strength is considered. This ratio is a good indicator for the material failure mode that is to be expected at an interface. Interfaces that have low cleavage strength and high frictional strength are likely to fail in brittle cleavage, while high cleavage strength and low frictional strength is beneficial for gradual material flow and ductile failure. The results for $\tau /{\sigma }_{C}$ are plotted in Fig. 4(b), and we see very similar results compared to Fig. 4(a). Brittle materials (like Si, Ge, Be, or W) exhibit a stronger colouring than ductile ones (Au, Pt, Cu, Ti, …). Since both ratios are dimensionless, it is not surprising that the scales of the subfigures are equivalent. It should be mentioned that the data is computed for all considered interfaces and plotted only for the one with the lowest ratio, corresponding to Figs 2 and 3. The data for all considered interfaces is given in the supplementary information. If inspected closely, $\tau /{\sigma }_{C}$ in Fig. 4(b) correctly shows gold as the material with the best ductility, indicating that this method is superior to estimate the ductility of an interface than Δ*γ/γ*.

## Discussion

The panels (a) and (b) of Fig. 2 and (a) of Fig. 3 present similar trends among the elements, which we can trace back to the behaviour of the cohesive energy in elemental solids. For the simple *sp*-metals, the cohesive energy is low, due to the comparatively weak metallic bonding, which is leading to low adhesion energy and in turn also to low shear- and cleavage strength. Beryllium, with its large deviations from the free-electron behaviour and small atomic radius, is a well known counter-example^40^. For the *d*-metals, the cohesive energy is increasing until all 5 bonding states of the *d*-shell are filled, and decreasing afterwards, when the anti-bonding states are populated. This trend can be clearly observed for *γ*, $\tau$, and *σ*_*C*_.

This suggests a close relation between these three quantities, which is a very interesting result, since the computational effort for the calculation of these three material parameters is not equivalent. The adhesion is easiest to calculate for the systems we consider, since the position of the minimum is predetermined by the ideal bulk stacking of the system. For the ideal shear strength, one needs to compute the whole *γ*-surface and the MEP, which involves not only many more DFT calculations, but also interpolation between high-symmetry PES points and some algorithm to find the MEP. Analogous, the computation of *σ*_*C*_ relies on a significant number of DFT calculations and some fitting routines. Thus, finding functional relations $\tau =f(\gamma )$ and $\sigma =g(\gamma )$ would considerably reduce the computational workload to estimate the ideal strength of a material.

**Figure 6:**
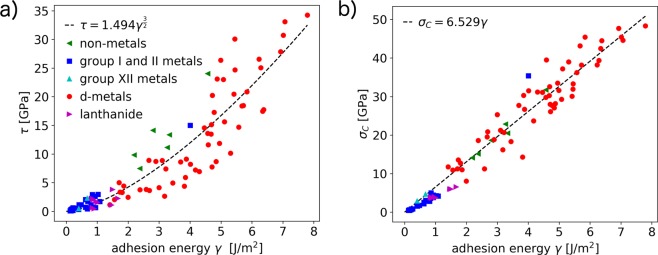
Ideal shear strength along the MEP $\tau$ (**a**) and ideal cleavage strength *σ*_*C*_ (**b**) vs adhesion *γ* for all investigated interfaces. The data are fitted to a power law with exponent 3/2 in (**a**) and with a linear function in (**b**). These least square fits are shown as dashed lines.

These functional relations are plotted in Fig. 6. Indeed the data cluster around simple functions: For the ideal shear strength we observe a power law with exponent 3/2, while the ideal cleavage strength is linearly dependent on the adhesion. The results of Fig. 6(a) allow us to confirm the correlation between adhesion and friction of clean, flat interfaces which we established in our previous work^41^.

The linear connection between the adhesion *γ* and the ideal cleavage strength *σ*_*C*_ is not too surprising if one recalls Eq. 2 where the restoring force is a linear function of the cleavage energy *E*_*C*_. For most materials *E*_*C*_ is close to the adhesion *γ*, unless the surfaces relax significantly from the bulk positions. Since the critical length $\ell$, which enters in the denominator of Eq. 2, varies only between 0.42 Å and 1.28 Å, its variation perturbs the linear fit only slightly.

The fitted proportionality constants are ∼1.5 skg^2/3^ nm^−1^ for the power law and ∼6.5 nm^−1^ for the linear relationship. It should be noted that while these functional relations allow to estimate the shear and cleavage strength of materials based on their adhesion, the scattering of the data around the fitted curve has to be considered. The average deviation of the data to the curves is 2.4 GPa for $\tau$, and 2.3 GPa for *σ*_*C*_, but considering that the absolute values of these data reach over 30 GPa (for $\tau$) and around 50 GPa (for *σ*_*C*_), the predictive power of the fits are respectable. We clearly have shown that the adhesion is an important descriptor of a given solid material and can be used to estimate material characteristics which are much harder to compute.

As mentioned in the introduction, we compute the strength of homogenous interfaces under shear and tensile strain using high throughput methods developed for interfaces. Our approach is based on analysis of the *γ*-surface and can be easily generalized to more complex interface situations, including adatoms, defects and heterogeneous slabs. The ideal strength of can be calculated in a different way^42,43^: A bulk cell is put under more and more shear or tensile strain and the resulting stress on the system is computed. At each strain value the atomic positions are fully relaxed. The result is a stress-strain curve and the ideal strength of the material is equivalent to the maximum of this curve. In contrast to our approach, elastic and phonon instabilities can also be considered within this method, although they are often neglected^44^.

This approach is quite different from our methodology, which is especially tailored to interfaces and was developed in the context of adhesive friction^16^. However, for systems where the interplanar slip between each pair of crystal planes is sufficiently decoupled from the slip between other pairs, the maximum resistance to slip should be similar to the shear strength of a bulk system calculated by continuous unit cell deformations and including atomic relaxations^14^. In the case of tensile strain the situation is different, as is discussed in detail in the Supplementary Information. As mentioned before our method implicitly assumes brittle cleavage along the investigated plane, while the stepwise straining of a bulk cell perpendicular to this plane allows ductile failure.

In Table 1 we compare our data to literature values for some quite common materials of all considered lattice types. The cleavage strength data are computed analogously to our approach with rigid body separation and fits to UBER. We are not aware of any previous first principles calculation of ideal shear strength of solid interfaces with our method, so we compare *τ* along the MEP to the shear strength computed by quasi-continuous unit cell deformations. Initially, we have to note that traditionally ideal shear strength is computed only for the ‘easy’ planes, e.g. 111 for fcc and 110 for bcc, with other data being hard to find. This highlights the importance of our work for creating a complete database for shear- and cleavage-strengths, since to date not much data is available for even the common materials of Table 1. The cleavage strength data is in excellent agreement with the literature across the board, with small deviations rooted in the use of different density functionals. Where we could get literature data for the ideal shear strength we notice generally good agreement between our data and the literature, apart from the materials with highly directional bonds, such as Si and Ge, where our approach is expected to differ from quasi-continuous cell deformations^14^. Experimental data are marked with a * in Table 1. As was to be expected, the ideal, intrinsic values computed in this and other works are usually about one order of magnitude larger than the experimental results of real materials.

**Table 1:** Comparison of some results from this work (Calc.) to values from literature (Lit.). The first three materials are bcc, followed by two examples of the diamond lattice, three fcc metals, and three hcp crystals.

	100				110				111/0001			
	*τ*[GPa]		*σ*[GPa]		*τ*[GPa]		*σ*[GPa]		*τ*[GPa]		*σ*[GPa]	
	Calc.	Lit.	Calc.	Lit.	Calc.	Lit.	Calc.	Lit.	Calc.	Lit.	Calc.	Lit.
Fe	26.4	—	31.6	—	11.9	7.5–8.14^43^, 3.56*^44^	33.5	—	24.7	—	33.3	13.1*^44^
Mo	26.6	—	38.2	23^53^	14.7	15.1–16.1^43^	36.2	38.8^54^	25.1	—	39.4	—
W	34.2	—	48.4	47–48^52,54^	17.7	17.1–20.8^43,44^	44.5	44^52^	27.9	—	47.7	46^52^
Si	24.0	—	31.8	—	11.1	—	22.9	—	13.4	6.5–9.62^43^	20.5	21^55^
Ge	14.1	—	18.6	—	7.5	—	15.2	—	9.8	4.5–5.2^43^	14.1	—
Al	4.0	—	12.0	12^55^	5.2	—	11.7	12^55^	2.1	1.85–3.5^43,44^	10.5	11-11.8^54,55^
Cu	8.9	—	19.5	1.5–1.74*^44^	8.7	0.8*^44^	18.8	1.59*^44^	3.8	2.16–5.3^43,44^, 0.46–0.61*^44^	18.6	19.6^54^, 1.25–1.84*^44^
Ni	14.6	—	31.1	33.2^54^	13.6	—	29.8	28.5^54^	5.9	5.05–6.4^43^	30.3	31.5^54^
Mg	—	—	—	—	—	—	—	—	0.7	1.8^56^	5.0	6.73^54^
Ti	—	—	—	—	—	—	—	—	4.4	—	14.3	—
Zn	—	—	—	—	—	—	—	—	2.3	2.1^56^	4.7	—

Experimental data are marked with a *, while all other data are from computations. Please note that in case of our data the symbol τ stands for the maximum resistance to slip. A table with our results for all considered materials can be found in the supplementary information.

In summary, we have presented a high-throughput DFT study on the adhesion and the ideal shear and cleavage strength of various lattice planes of solid elemental crystals. We have automatically computed tribological figures of merit for over one hundred different interfaces for elemental crystals in the fcc, bcc, hcp, and diamond structure. Our results are based on the detailed analysis of the *γ*-surface, which we calculate ab-initio using high symmetry points and interpolation via radial basis functions. By plotting the results in a periodic table, we observe clear trends in the data. Thus we show that the mechanical properties of elemental crystals indeed are closely related to their electronic structure. This confirms the notion that adhesive friction is rooted in the chemical interactions at the interface and ultimately governed by quantum mechanics^41,45^. We find good agreement of our data with previously published cleavage strength values. We are not aware of any previous first principle calculation of the ideal shear strength of solid interfaces, thus we compare our result to the (few) shear strengths calculated from first principles for bulk cells in the literature. The comparison suggest that for materials with no directional bonds our result can provide close estimates and thus significantly extend the available data for this important material parameter. We have also found that the adhesion is a good descriptor for the much harder to compute shear and cleavage strength values for a given interface. The ideal shear strength along the minimum energy path is proportional to the adhesion to the power of 3/2, while the cleavage strength is directly proportional to the adhesion. The ratio between the ideal shear and the cleavage strengths is provided as indicator of the material failure mode that is to be expected at an interface. Interfaces that have high shear strength/cleavage strength ratio are likely to fail in brittle cleavage, while low value of the ratio characterize ductile failure. We believe that the data we computed may become very important as input parameters for large scale continuum simulations and can also serve as benchmarks for classical atomistic models. We make all curated and raw data available in an online repository for direct access and for further analysis.

## Methods

All results in this work are calculated within the *Automated Interactive Infrastructure and Database for Computational Science* (AiiDA^2^) framework. We have developed an high-throughput AiiDA workflow to calculate interface data automatically with density functional theory (DFT), which we have described in detail previously^21^. The main capabilities are restated here briefly:

- All computational and structural parameters (energy cutoffs, k-point-grids, and lattice parameters) are converged automatically.
- The potential energy surface (PES, also called *γ*-surface or generalized stacking fault energy) is computed by interpolating between calculated high symmetry points. The minimum energy path (MEP), which is the path through the PES with the least resistance to sliding, is then computed. The minimum of the PES is equivalent to the adhesion *γ* of the interface.

- The ideal shear strength of the interface is determined along the MEP and some high symmetry directions of the lattice in question.
- We obtain the perpendicular energy profile *γ*(*z*) by rigidly separating the two slabs forming the interface in discrete steps and computing the interaction energy.

All computations have been performed with the quantum espresso package^46^, using ultrasoft PBE-GGA pseudopotentials^47^, which are well suited to describe the chemically bonded materials discussed in this paper. It should however be mentioned that the option to include van der Waals interactions in the form of the semi-empirical Grimme method^48^ (e.g. for the computation of graphite) is possible by simply setting a flag in the input file. This method has been proven to provide good results in previous studies on layered systems^38,49^. In general the choice of exchange-correlation functional will influence the results by up to several percentage points. For example the LDA is known to overbind, but gives better magnetic moments, while PBE usually slightly underbinds with average errors which are lower than for LDA. Overall our choice of PBE is routed in the fact that it is robust functional that performs reasonably well for all systems, which is an excellent behaviour for a high throughput study. It is also a very well known functional and a lot of people know its tendencies, strengths, and weaknesses.

For a more detailed description of the workflow and some code parts, the reader is referred to our previous paper^21^. In the following we will describe the improvements made to the workflow since we published that paper.

### Improvements to the workflow

While the workflow we used was essentially the same as described in our recent paper^21^, three conceptual improvements have been made:

First, the convergence of the kinetic energy cutoff of the wavefunctions ${E}_{{\rm{cut}}}^{{\rm{WF}}}$ is no longer achieved by converging the total energy difference between the input structure at two different volumes for increasing ${E}_{{\rm{cut}}}^{{\rm{WF}}}$. Instead, we rescale the input structure to 5 different volumes, ranging from 90% to 110%, of the volume corresponding to the trial lattice parameter. The total energies of these 5 structures are then fitted to a Birch-Murnaghan equation of state to obtain the lattice parameter^50^. Now, we increase ${E}_{{\rm{cut}}}^{{\rm{WF}}}$ in steps of 5 Ry (starting at 20 Ry) and repeat the process until the lattice parameter is converged. This eliminates some stability problems we encountered in converging the lattice parameter using Newton’s method for some materials. Furthermore, we converge the lattice parameter and *E*_cut_ at the same time and obtain also a value for the bulk modulus of the material. The cutoff for the charge density ${E}_{{\rm{cut}}}^{{\rm{CD}}}$ is obtained as before, by multiplying ${E}_{{\rm{cut}}}^{{\rm{WF}}}$ by a user-specified factor which defaults to 12.

The second difference is that we implemented the (0001) interface (basal plane) of hexagonal close packed (hcp) structures. Since most elements in the periodic table that crystallize in the hcp structure do not exhibit the ideal *c*/*a* ratio of $\sqrt{8/3}$, this is initially optimized by a variable-cell relaxation using a very dense k-point mesh and extremely high values for the charge density cutoffs (${E}_{{\rm{c}}{\rm{u}}{\rm{t}}}^{{\rm{W}}{\rm{F}}}=75\,{\rm{R}}{\rm{y}}$, ${E}_{{\rm{c}}{\rm{u}}{\rm{t}}}^{{\rm{C}}{\rm{D}}}=1000\,{\rm{R}}{\rm{y}}$) to ensure correct forces and a negligible influence of Pulay stress. Once the *c/a* ratio is determined, the lattice parameter and ${E}_{{\rm{cut}}}^{{\rm{WF}}}$ are converged as described previously.

The third change regards the perpendicular potential profile *γ*(*z*). We used to fit the data points with splines to provide a smooth curve and guide the eye. In this work we have fitted the data in the attractive regime with the UBER relation^51^ (Eq. 1). This avoids ambiguities in the exact fits (type of splines and boundary conditions) and connects our data with the established rigid body separation model for brittle fracture^32,52^.

## Supplementary information

Supplementary Information

## Data Availability

The interested reader is invited to download the current version of the workflow from the homepage of our group: http://www.tribchem.it/workflow/. All data described and analysed in this publication is available in a large, nested python dictionary provided as a supplementary file. We also prepared some example python scripts that show how to access the data.
