# Supplementary material for: High-throughput screening of the static friction and ideal cleavage strength of solid interfaces
Source: Sci Rep. 2019 Nov 19;9:17062. doi: 10.1038/s41598-019-49907-2 (PMC6863866; doi:10.1038/s41598-019-49907-2)
Supplement: Supplementary file 1 — Supplementary Information [file 41598_2019_49907_MOESM1_ESM.pdf]

Supplementary information for

High-throughput screening of the cleavage and shear strengths of solid interfaces

Michael Wolloch[1,2], Gabriele Losi[1], Mauro Ferrario[1], and M. Clelia Righi[1,3,*]

[1]Dipartimento di Scienze Fisiche, Informatiche e Matematiche, Università di Modena e Reggio Emilia, Via Campi 213/A, 41125 Modena, Italy
[2]Faculty of Physics, University of Vienna, Währinger Strasse 17, 1090 Vienna, Austria
[3]CNR-Institute of Nanoscience, S3 Center, Via Campi 213/A, 41125 Modena, Italy
[*]mcrighi@unimore.it

Computed high level data for all considered interfaces

Table S1 shows the most important results we computed for all considered interfaces. In table S2 the different values for the SFF/A are listed in detail. Together with the SFF/A along the MEP we present the values along two high symmetry directions which are orthogonal to each other. For some interfaces the differences are considerable, and the ratio between the two numbers may be viewed as a measure for the frictional anisotropy of the sliding system.

In table S3 we compare our data for the cleavage strength σ_C for some example materials (the same as used in the table in the main paper) to previously calculated data for the ultimate tensile strengthσ_{ut} of these materials. We note that the cleavage strength is fitting well for the more brittle materials at surfaces that are cleavage planes (e.g. W(110) and Si(111)) as expected. For more ductile materials or other lattice planes the materials will rather fail through slip along a lattice plane which is at an angle to the investigated interface, or through the creation and slip of dislocations. In this cases the computed cleavage strength is considerable higher than the ultimate tensile strength.

We remind the reader that a larger amount of information, including computational parameters and raw data, is available as a nested python dictionary alongside a number of python scripts that demonstrate how one can use this data for further analysis.

Details about the rigid separation model and the UBER fits

The rigid separation model might seem like a crude approximation to a material failing under tensile strain. However, it is specifically designed to model brittle fracture, for which it performs well[4].

Including relaxations of atomic layers perpendicular to the interface plane is possible, but one has to take several finite size effects into account to achieve a physically consistent solution[5,6]: When elastic effects are considered, the surfaces should separate when the strain energy exceeds the cleavage energy. However, the strain energy per unit area decreases with the system size, as does the critical stress wich is linearly dependent on it for small enough strains. Thus, if the crystal is large enough, both strain energy and critical stress go to zero, and the crystal is not stable with respect to uniaxial strain. To overcome this unphysical result, one has to further consider that the energy barrier for the separation, which can be calculated by transition state theory, increases with the square root of the number of layers in the system[6]. To summarize, the correct treatment of relaxations while separating interfaces under tensile strain is complex, and one needs to investigate system size dependent energies rather than stresses. On the other hand, the simple rigid body separation model we employ is not size dependent, provides reliable upper limits for the ideal tensile strengths of materials[6] and was shown to be well justified by comparison to a simulation of a crack tip under mode 1 loading[7].

In Fig. S3 we plot the UBER fits for each lattice type. (For bcc and fcc lattices only the curves with the lowest cleavage strength σ_C are plotted to avoid cluttering of the plots.) The color coding corresponds to the cleavage energy E_C, with dark blue lines showing the lowest values and dark red the highest.

References

1. Pokluda, J., Černý, M., Šob, M. & Umeno, Y. Ab initio calculations of mechanical properties: Methods and applications. *Prog. Mater. Sci.* **73**, 127 – 158, DOI: 10.1016/j.pmatsci.2015.04.001 (2015).

Table S1. We report the lattice parameter a (Å), the adhesion energy (J/m^2), the static friction force per unit area (SFF/A) along the MEP (GPa), and the cleavage strength (GPa) for all the analysed materials and interfaces.

			100			110			111/0001		
Z	Element	a	$\gamma_{\min}$	τ_F^{MEP}	σ_C	$\gamma_{\min}$	τ_F^{MEP}	σ_C	$\gamma_{\min}$	τ_F^{MEP}	σ_C
3	Li	3.47	0.85	2.93	3.6	0.93	0.66	4.46	1.03	2.95	4.25
4	Be	2.28							4.01	15.01	35.4
11	Na	4.26	0.41	1.15	1.57	0.4	0.33	1.79	0.48	1.14	1.78
12	Mg	3.32							0.86	0.75	4.99
13	Al	4.04	1.84	4.0	11.96	2.13	5.22	11.72	1.52	2.06	10.51
14	Si	5.47	4.57	24.03	31.76	3.26	11.13	22.88	3.33	13.35	20.48
19	K	5.28	0.21	0.63	0.7	0.21	0.16	0.85	0.26	0.55	0.86
20	Ca	5.47	0.93	2.61	3.82	1.08	1.72	4.14	0.95	0.65	4.14
21	Sc	3.32							2.58	3.82	11.22
22	Ti	2.94							3.81	4.38	14.29
23	V	2.99	4.88	23.09	28.16	4.84	8.7	27.06	5.42	20.46	30.06
24	Cr	2.86	7.07	33.1	44.61	6.35	17.46	42.47	7.01	30.73	45.44
26	Fe	2.83	4.99	26.36	31.58	4.95	11.86	33.48	5.45	24.68	33.31
27	Co	2.49							4.3	6.95	31.16
28	Ni	3.5	4.46	14.57	31.09	4.57	13.59	29.78	3.85	5.92	30.32
29	Cu	3.63	2.67	8.9	19.53	2.9	8.71	18.82	2.38	3.76	18.58
30	Zn	2.65							0.67	2.29	4.68
32	Ge	5.76	2.8	14.12	18.64	2.38	7.46	15.15	2.19	9.82	14.09
37	Rb	5.68	0.17	0.4	0.58	0.15	0.12	0.61	0.19	0.43	0.62
38	Sr	6.04	0.69	1.73	2.87	0.8	1.14	3.05	0.68	0.44	3.07
39	Y	3.65							2.0	2.47	8.06
40	Zr	3.24							3.18	2.64	16.18
41	Nb	3.28	4.72	20.24	26.1	4.17	7.22	23.71	4.78	17.27	27.65
42	Mo	3.15	6.23	26.56	38.24	5.57	14.71	36.25	6.28	25.07	39.4
44	Ru	2.72							5.11	15.62	37.21
45	Rh	3.83	4.69	15.12	32.59	4.68	13.47	30.26	4.01	8.23	31.51
46	Pd	3.95	3.1	8.86	21.24	3.22	7.4	20.68	2.68	3.66	20.85
47	Ag	4.15	1.7	5.0	11.25	1.86	4.43	11.01	1.56	2.07	10.93
48	Cd	3.04							0.4	0.5	2.91
50	Sn	6.66	1.75	5.85	10.68	1.26	3.61	9.53	1.22	3.81	8.19
55	Cs	6.16	0.14	0.31	0.46	0.12	0.09	0.48	0.16	0.09	0.48
56	Ba	5.0	0.62	2.06	2.16	0.62	0.71	2.54	0.81	2.23	2.82
58	Ce	5.27	1.49	3.8	5.96	1.67	2.25	6.51	1.46	1.1	5.99
63	Eu	5.54	0.87	2.02	3.61	1.0	1.53	3.75	0.9	0.43	3.7
70	Yb	5.5	0.83	2.24	3.73	0.96	1.75	3.94	0.84	0.63	3.91
72	Hf	3.2							3.44	4.96	18.33
73	Ta	3.32	5.08	22.99	29.29	4.71	7.74	28.0	5.45	30.08	31.92
74	W	3.19	7.79	34.23	48.37	6.38	17.74	44.5	6.93	27.86	47.69
75	Re	2.77							5.31	10.15	39.0
76	Os	2.75							5.84	20.85	45.44
77	Ir	3.88	5.68	18.4	43.17	5.72	18.46	38.16	4.59	11.63	39.5
78	Pt	3.98	3.7	8.61	27.71	3.88	9.13	26.7	2.99	4.13	25.29
79	Au	4.15	1.74	3.38	13.55	1.8	3.29	12.73	1.41	1.16	11.75
82	Pb	5.04	0.53	1.12	3.28	0.78	1.67	4.78	0.5	0.64	4.0

Table S2. We report the static friction force per unit area along the MEP and two high symmetry directions (GPa), as well as their ratios, for all the analysed materials and interfaces.

		100				110				111/0001			
Z	Element	τ_F^{100}	τ_F^{010}	τ_F^{MEP}	$\tau_F^{100}/\tau_F^{010}$	τ_F^{001}	$\tau_F^{\bar{1}10}$	τ_F^{MEP}	$\tau_F^{001}/\tau_F^{\bar{1}10}$	τ_F^{101}	τ_F^{121}	τ_F^{MEP}	$\tau_F^{101}/\tau_F^{121}$
3	Li	2.93	2.93	2.93	1.0	1.6	1.15	0.66	1.38	2.84	2.95	2.95	0.95
4	Be									20.45	28.53	15.01	0.72
11	Na	1.15	1.15	1.15	1.0	0.79	0.58	0.33	1.36	1.19	1.14	1.14	0.98
12	Mg									1.45	2.52	0.75	0.58
13	Al	4.01	4.01	4.0	1.0	5.22	8.18	5.22	0.64	3.08	2.06	2.06	0.82
14	Si	26.83	24.03	24.03	1.12	11.13	24.28	11.13	0.46	14.14	13.35	13.35	1.07
19	K	0.63	0.63	0.63	1.0	0.38	0.27	0.16	1.4	0.59	0.55	0.55	0.96
20	Ca	2.61	2.61	2.61	1.0	1.72	4.36	1.72	0.39	1.26	0.65	0.65	0.61
21	Sc									4.7	5.92	3.82	0.79
22	Ti									4.99	5.81	4.38	0.86
23	V	23.1	23.1	23.09	1.0	13.6	9.64	8.7	1.41	22.36	20.46	20.46	0.83
24	Cr	33.1	33.1	33.1	1.0	27.63	17.72	17.46	1.56	34.48	30.73	30.73	0.82
26	Fe	26.36	26.36	26.36	1.0	21.95	13.44	11.86	1.63	26.82	24.68	24.68	1.09
27	Co									11.17	18.32	6.95	0.61
28	Ni	14.57	14.57	14.57	1.0	13.6	23.2	13.59	0.59	9.6	5.92	5.92	0.71
29	Cu	8.92	8.92	8.9	1.0	8.71	14.58	8.71	0.6	6.09	3.76	3.76	0.69
30	Zn									3.6	4.26	2.29	0.85
32	Ge	14.15	16.35	14.12	0.87	7.46	11.90	7.46	0.04	10.43	9.82	9.82	1.08
37	Rb	0.4	0.4	0.4	1.0	0.27	0.2	0.12	1.35	0.43	0.43	0.43	0.97
38	Sr	1.73	1.73	1.73	1.0	1.14	2.94	1.14	0.39	0.87	0.44	0.44	0.58
39	Y									3.25	4.36	2.47	0.75
40	Zr									4.19	5.93	2.64	0.71
41	Nb	20.28	20.28	20.24	1.0	12.76	8.33	7.22	1.53	20.98	17.27	17.27	0.77
42	Mo	26.56	26.56	26.56	1.0	23.13	15.38	14.71	1.5	38.69	25.07	25.07	1.03
44	Ru									18.57	22.69	15.62	0.82
45	Rh	15.13	15.13	15.12	1.0	13.48	20.62	13.47	0.65	11.12	8.23	8.23	0.88
46	Pd	8.88	8.88	8.86	1.0	7.41	14.73	7.4	0.5	5.61	3.66	3.66	0.82
47	Ag	5.01	5.01	5.0	1.0	4.44	7.98	4.43	0.56	3.31	2.07	2.07	0.65
48	Cd									1.0	1.56	0.5	0.64
50	Sn	5.87	10.2	5.85	0.58	3.61	5.86	3.61	0.62	4.15	3.81	3.81	1.1
55	Cs	0.31	0.31	0.31	1.0	0.21	0.15	0.09	1.35	0.44	0.09	0.09	1.11
56	Ba	2.06	2.06	2.06	1.0	1.51	0.96	0.71	1.57	2.18	2.23	2.23	0.92
58	Ce	3.81	3.81	3.8	1.0	2.25	5.66	2.25	0.4	1.48	1.1	1.1	0.76
63	Eu	2.02	2.02	2.02	1.0	1.53	4.14	1.53	0.37	0.94	0.43	0.43	0.53
70	Yb	2.25	2.25	2.24	1.0	1.75	3.81	1.75	0.46	1.27	0.63	0.63	0.59
72	Hf									5.89	7.12	4.96	0.83
73	Ta	23.01	23.01	22.99	1.0	15.75	9.47	7.74	1.66	30.97	30.08	30.08	1.03
74	W	34.25	34.25	34.23	1.0	30.47	21.38	17.74	1.42	31.88	27.86	27.86	1.14
75	Re									15.31	27.74	10.15	0.55
76	Os									24.2	28.96	20.85	0.84
77	Ir	18.42	18.42	18.4	1.0	18.47	22.87	18.46	0.81	15.06	11.63	11.63	1.03
78	Pt	8.66	8.66	8.61	1.0	9.14	15.61	9.13	0.59	5.86	4.13	4.13	1.38
79	Au	3.38	3.38	3.38	1.0	3.3	5.97	3.29	0.55	2.11	1.16	1.16	0.65
82	Pb	1.13	1.13	1.12	1.0	1.67	2.67	1.67	0.63	1.01	0.64	0.64	0.93

	100		110		111	
	σ_C[GPa]	σ_{ut}[GPa]	σ_C[GPa]	σ_{ut}[GPa]	σ_C[GPa]	σ_{ut}[GPa]
Fe	31.6	10.6–14.2[1]	33.5	16.7–33[1]	33.3	19.3–27.7[1]
Mo	38.2	26.7–28.8[1]	36.2	32.6–40.7[1]	39.4	28.4–29.9[1]
W	48.4	27.7–29.5[1]	44.5	35.9–54.3[1]	47.7	39–40.1[1]
Si	31.8	26.3–27.5[1]	22.9	17–17.9[1]	20.5	21–24.8[1]
Ge	18.6	16.4–16.8[1]	15.2	11.8[1]	14.1	14–14.6[1]
Al	12.0	9–13.1[1]	11.7	4.5–4.92[1]	10.5	8.8–11.52[1,2]
Cu	19.5	9.3–36[1,3]	18.8	4.6–31[1]	18.6	7.5–29[1]
Ni	31.1	18.3–36.1[1]	29.8	9.1–10.5[1]	30.3	15.4–34.1[1]

Table S3. Comparison of some results (the same materials as in the table in the main paper) from this work for the ideal cleavage strength (σ_C) to previously calculated values of uniaxial tensile strength (σ_{ut}) from previous works. Data are split into 3 groups: bcc, diamond, fcc crystals.

2. Li, W. & Wang, T. Ab initio investigation of the elasticity and stability of aluminum. *J. Physics: Condens. Matter* **10**, 9889–9904, DOI: 10.1088/0953-8984/10/43/033 (1998).

3. Pokluda, J., Černý, M., Šandera, P. & Šob, M. Calculations of theoretical strength: State of the art and history. *J. Comput. Mater. Des.* **11**, 1–28, DOI: 10.1007/s10820-004-4567-2 (2004).

4. Lazar, P., Podloucky, R. & Wolf, W. Correlating elasticity and cleavage. *Appl. Phys. Lett.* **87**, 261910, DOI: 10.1063/1.2149988 (2005).

5. Lazar, P. & Podloucky, R. Cleavage fracture of a crystal: Density functional theory calculations based on a model which includes structural relaxations. *Phys. Rev. B* **78**, 104114, DOI: 10.1103/PhysRevB.78.104114 (2008).

6. Elsner, B. A. M. & Müller, S. Size effects and strain localization in atomic-scale cleavage modeling. *J. Physics: Condens. Matter* **27**, 345002, DOI: 10.1088/0953-8984/27/34/345002 (2015).

7. Möller, J. J., Bitzek, E., Janisch, R., ul Hassan, H. & Hartmaier, A. Fracture ab initio: A force-based scaling law for atomistically informed continuum models. *J. Mater. Res.* **33**, 3750–3761, DOI: 10.1557/jmr.2018.384 (2018).

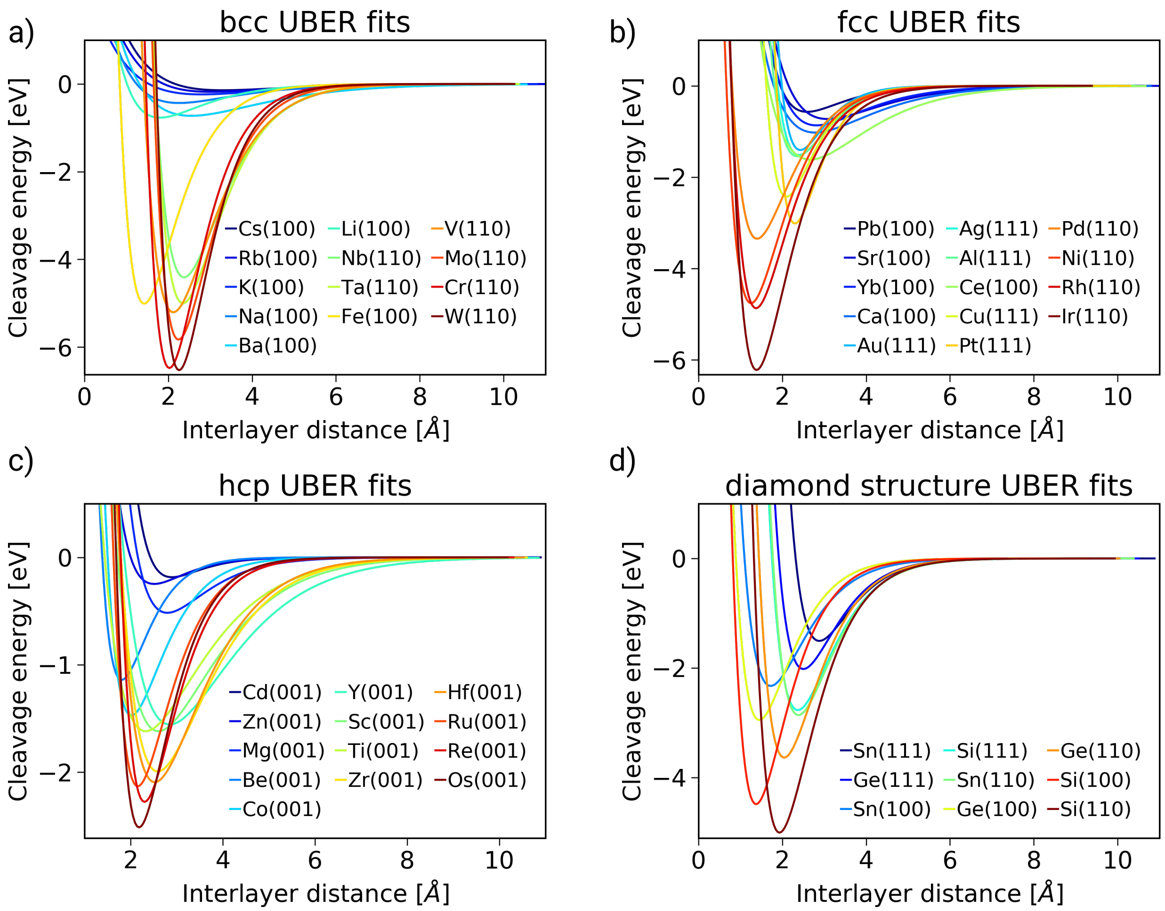

Figure S3. Universal binding energy relation fits to the attractive regime of the computed $\gamma(z)$ data. For the lattice types a) bcc and b) fcc only the interface planes with the smallest cleavage strengths are shown since the graphs would be too cluttered otherwise. For c) hcp and d) diamond lattices, we show all avaiable data. Note the different scales on the subplots.
